# Intra-osseous Co-transplantation of CD34-selected Umbilical Cord Blood and Mesenchymal Stromal Cells

**DOI:** 10.15761/HMO.1000105

**Published:** 2016-10-20

**Authors:** Leland Metheny, Saada Eid, Karen Lingas, Jane Reese, Howard Meyerson, Alexander Tong, Marcos de Lima, Alex Y Huang

**Affiliations:** 1 Stem Cell Transplant Program, University Hospitals Case Medical Center and Case Western Reserve University, Cleveland, OH 44106, USA; 2 Case Comprehensive Cancer Center, Case Western Reserve University School of Medicine, Cleveland, OH 44106, USA; 3 Division of Pediatric Hematology/Oncology, Department of Pediatrics, Case Western Reserve University School of Medicine; Angie Fowler AYA Cancer Institute, University Hospitals Rainbow Babies & Children's Hospital, Cleveland, OH 44106, USA; 4 Department of Pathology, Case Western Reserve University School of Medicine; University Hospitals Case Medical Center, Cleveland, OH 44106, USA

**Keywords:** cord blood stem cell transplantatio, mesenchymal stromal cells, hematopoietic stem cell transplantation

## Abstract

Human mesenchymal stromal cells (MSC) have been shown to support the growth and differentiation of hematopoietic stem cells (HSC). We hypothesized that intra-osseous (IO) co-transplantation of MSC and umbilical cord blood (UCB) may be effective in improving early HSC engraftment, as IO transplantation has been demonstrated to enhance UCB engraftment in NOD SCID-gamma (NSG) mice. Following non-lethal irradiation (300rads), 6 groups of NSG mice were studied: 1) intravenous (IV) UCB CD34^+^ cells, 2) IV UCB CD34^+^ cells and MSC, 3) IO UCB CD34^+^ cells, 4) IO UCB CD34^+^ cells and IO MSC, 5) IO UCB CD34^+^ cells and IV MSC, and 6) IV UCB CD34^+^ and IO MSC. Analysis of human-derived CD45^+^, CD3^+^, and CD19^+^ cells 6 weeks following transplant revealed the highest level of engraftment in the IO UCB plus IO MSC cohort. Bone marrow analysis of human CD13 and CD14 markers revealed no significant difference between cohorts. We observed that IO MSC and UCB co-transplantation led to superior engraftment of CD45^+^, CD3^+^ and CD19^+^ lineage cells in the bone marrow at 6 weeks as compared with the IV UCB cohort controls. Our data suggests that IO co-transplantation of MSC and UCB facilitates human HSC engraftment in NSG mice.

## Introduction

Umbilical cord blood (UCB) is rich in hematopoietic stem cells (HSC) and it has been used as a graft source for both hematopoietic stem cell transplant (SCT) patients and murine models of transplant [1,2]. Usually, UCB is given after conditioning chemotherapy or radiation through intravenous (IV) infusion. However, due to delayed or sub-optimal engraftment in both mice and humans, other methods beyond IV infusion have been proposed. These include intra-osseous infusion, co-infusion of the UCB with mesenchymal stromal cells (MSC), or ex vivo expansion of UCB prior to infusion [3-5]. Here we report our data on intra-osseous co-infusion of UCB and MSC in a murine model of transplant.

It has been shown that a large percentage of HSCs, when introduced IV, do not reach the bone marrow niche [6,7]. Cui *et al.* demonstrated that when fluorescently labelled donor bone marrow cells are injected IV in an irradiated syngeneic mouse model, only 1-2% of donor HSCs reached the bone marrow [6]. In mouse models, direct IO injection of HSC improves overall engraftment, possibly by bypassing potential “trapping” sites in multiple organs including the lung, liver, spleen and kidney [8-10]. In an experiment performed by Castello *et al*. direct IO inoculation of UCB resulted in higher engraftment rates when compared to IV UCB injection 30 days post transplantation, as measured by the abundance of human myeloid cells, identified by CD45^+^ cells within the bone marrow [9].

The bone marrow microenvironment provides an important niche for the proliferation and differentiation of HSC [11-14]. Human MSC have been shown to support the growth and differentiation of HSC [15]. Bone marrow MSC support hematopoietic growth through a number of mechanisms including the production of IL-6, IL-11, leukemia inhibitory factor, stem cell factor and thrombopoietin [16,17]. MSC also aid in the homing and migration of HSC to the bone marrow [18]. Furthermore, the addition of MSC *ex vivo* to UCB cultures results in significant expansion of the hematopoietic stem (CD34^+^) cell pool. This method of *ex vivo* expansion has been used to increase the cell dosages of UCB grafts in patients undergoing SCT [12].

MSCs have also been co-infused with CD34-selected human UCB IV in a murine model of hematopoietic stem cell transplant [19]. In this model, mice transplanted with CD34 selected UCB and Stro-1^-^ MSCs exhibited significantly greater human hematopoietic engraftment in the bone marrow, spleen and blood (CD45%: 48, 35, and 14, respectively) when compared to mice transplanted with UCB alone (CD45%: 27, 15, and 4, respectively) at 12 weeks following transplantation. These data formed the basis for the approach of using a combination of MSC and UCB co-transplantation IV in clinical trials, which were found to be safe [20].

Carrancio *et al*. demonstrated that in a mouse transplantation model, co-infusion of human MSCs and human CD34^+^ UBC enhanced the engraftment of myeloid compartment when compared to controls [21]. Mice co-transplanted with MSC and UCB by either the IV or IO route had greater B-cell (CD19^+^) and myeloid (CD13^+^) chimerism at 3 weeks following transplantation. Furthermore, mice transplanted with IO UCB and IO MSC had the greatest rate of human engraftment (CD45^+^) in the injected femur. Based on the data above, we hypothesized that co-transplantation of MSC and UCB via direct IO route would further improve early human engraftment in a mouse transplantation model.

## Methods

### Mice

Non-Obese Diabetic- Severe Combined Immunodeficiency-IL2Rgammanull (NSG) mice were from breeding pairs originally purchased from Jackson Laboratories. NSG were bred in a pathogen-free unit and maintained in sterile cages.

### Human MSCs

Human MSC (CD105^+^ CD73^+^ CD45^-^ CD14^-^) were obtained from bone marrow donors using Percoll gradient isolation and culture-expanded to a homogeneous population under approved protocols in the National Center for Regenerative Medicine/Seidman Cancer Center Cellular Therapy Lab. MSCs were preserved in DMSO and when needed were thawed and re-suspended at the appropriate concentration per cohort.

### CD34^+^ UCB cell isolation

Cord blood units were received from the Cleveland Cord Blood Center. Each unit was diluted 1:3 with Phosphate Buffered Saline (PBS) + 0.5% Human Serum Albumin (HSA) and layered onto Ficoll Paque PLUS to isolate the mononuclear cells by density gradient. After a cell count and washes, the mononuclear cells were labeled per protocol using the Miltenyi Biotec CD34 Microbead Kit. The CD34 cells were then isolated using an LS column in a magnet and washed three times with MACS buffer. The CD34 cells from both cord blood units were combined, counted, and re-suspended in PBS for injection into mice.

### Murine transplantation

Female Nude Obese Diabetic Severe Combined Immunodeficiency (NSG) mice aged 8 to 12 weeks were purchased from Jackson Laboratory (Bar Harbor, ME). Prior to SCT, recipient mice were ear punched for individual identification. Mice received allogeneic SCT using CD34 selected human umbilical cord blood. NSG mice received 300 cGy TBI immediately prior to receiving 5 × 10^5^ CD34^+^ selected cells from human umbilical cord blood with or without 1 × 10^6^ human MSC.

Six groups of NSG mice were studied: 1) intravenous (IV) 5×10^5^ UCB CD34^+^ cells, 2) IV 5 × 10^5^ UCB CD34^+^ cells and 1 × 10^6^ MSC, 3) IO 5 × 10^5^ UCB CD34^+^ cells, 4) IO 5 × 10^5^ UCB CD34+ cells and IO 1 × 10^6^ MSC, 5) IO 5×10^5^ UCB CD34^+^ cells and IV 1 × 10^6^ MSC, and 6) IV 5×10^5^ UCB CD34+ and IO 1 × 10^6^ MSC. MSC dose was arbitrarily set at 2:1 with UCB CD34^+^ cell dose. IV injections: cells were administered via tail vein injection suspended in a total volume of 200 μl. IO injections: Mice were anesthetized using fluorine gas, the left leg was shaved, the hair removed and betadine was applied to the skin. Cells were administered via bilateral tibia IO injections suspended in a total volume of 40 μl (20 μl in each tibia) using a 30-gauge needle. There were five mice in each cohort. Flow Cytometry: Peripheral blood samples from mice were analyzed on weeks 2, 4 and 6. Bone marrow tissue samples from mice were analyzed on week 6. Peripheral blood and bone marrow samples were stained for T cell markers, (CD3), myeloid markers (CD45, CD13, CD14) and with B-cell markers (CD19). Specific lineage markers (CD3, CD13, CD14 and CD19) were analyzed inside the human CD45^+^ population. All monoclonal antibodies (mAbs) were purchased from BD Biosciences Pharmingen (San Diego, CA) or eBioscience. At least 1 × 10^5^ events were analyzed per conjugated MAb stain condition. Data were analyzed using CFlow.

The liver, ileum, ascending colon, and right tibia were harvested. Organs were fixed in 10% buffered formalin, embedded in paraffin, cut into 5μm-thicksections, and stained with hematoxylin and eosin for histological examination. Slides were coded without reference to transplant group or treatment and reviewed in blinded fashion by a single pathologist. All animal studies were approved by the Institutional Animal Care and Use Committee (IACUC) at Case Western Reserve University (IACUC protocol 2015-0118).

### Histology

An independent hematopathologist, blinded to cohort characteristics, evaluated tibial sections, one from each mouse in every cohort (for a total of 5 per cohort), for myeloid-to-erythroid ratio, cellularity, and megakaryocyte percentages.

### Statistics

All values are expressed as the mean plus or minus standard error of the mean (± SEM). Statistical comparisons between groups were completed using Mann and Whitney test (nonparametric data).

## Results

### IO co-transplantation of CD34^+^ UCB and MSC improves overall HSC engraftment and enhances CD3^+^ T cell and CD19^+^ B cell recovery

**Figure 1:**
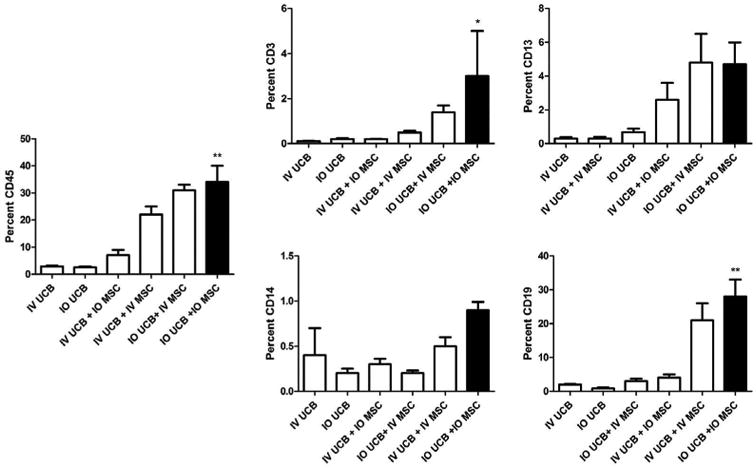
Percent CD45, CD3, CD13, CD14, CD19 in the right tibia bone marrow at 6 weeks post umbilical cord blood infusion. IV UCB = intravenous umbilical cord blood, IO UCB = intra-osseous umbilical cord blood, IV UCB + IV MSC = intravenous umbilical cord blood and mesenchymal stromal cells, IO UCB + IO MSC = intra-osseous umbilical cord blood and mesenchymal stromal cells, IV UCB + IO MSC = intravenous umbilical cord blood and intra-osseous mesenchymal stromal cells, IO UCB + IV MSC = intra-osseous umbilical cord blood and INTRAVENOUS mesenchymal stromal cells (* = p < 0.05; ** = p < 0.001). p-values as compared to the intravenous umbilical cord blood group.

Following irradiation, recipient mice were injected with MSC and CD34^+^ UCB via either the IV or IO route. At six weeks following transplantation, we assessed the degree of hematopoietic engraftment that can be traced to human CD34^+^ lineage by measuring cells that stained positive for human CD45 by flow cytometry. Cellular engraftment was significantly enhanced in the group receiving the IO co-transplantation of UCB and MSC as compared to IV UCB alone (34% vs. 2.9%, respectively; Figure 1 and Table 1; p < 0.001). Consistent with our hypothesis that administration of MSC improves overall UCB engraftment, we observed that any combination of UCB and MSC co-administration, irrespective of the mode of delivery of either the UCB or MSC, exhibited significantly improved HSC engraftment as compared to IV UCB injection alone. In particular, direct IO administration of UCB in the presence of either IO or IV MSC led to a trend towards improved engraftment at 6 weeks among CD45^+^ lineage cells as well as CD3^+^, CD13^+^, CD14^+^ and CD19^+^ cells (Table 1).

CD19^+^ B cell and CD3^+^ T cells recovery was significantly increased (p < 0.001 and p < 0.05, respectively) in the IO UCB and MSC co-transplantation cohort compared to IV UCB only cohort (B-cell 28% vs. 2%; T-cell 3% vs. 0.1%; Figure 1 and Table 1). Both the IV UCB/IV MSC and the IO UCB/IV MSC cohorts demonstrated significant improvement in engraftment over the IV UCB control. Specifically, CD19^+^ B cell recovery was significantly elevated in mice that received both UCB and MSC concurrently using the same route (IO or IV) of administration (28% vs. 21%, respectively) as compared to CD19^+^ B cell recovery when UCS and MSC were administered via different routes (IV/IO, 4%, IO/IV, 3%, respectively).

### Direct histologic evaluation of IO injected bone marrow

**Figure 2:**
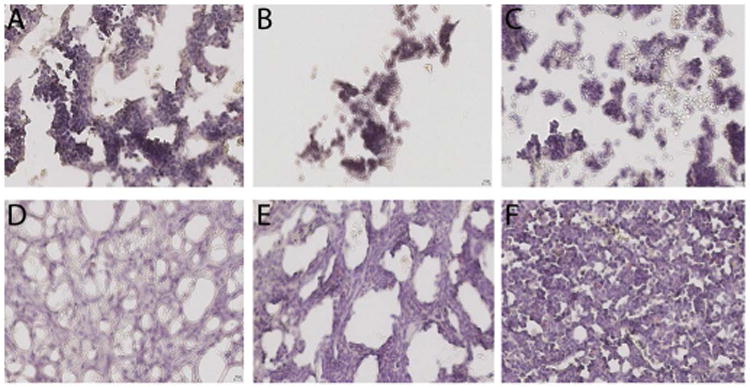
Histology of right tibia bone marrow from murine cohorts. A = intravenous (IV) umbilical cord blood (UCB), B = IV UBC and IV Mesenchymal Stromal Cells (MSC), C = intra-osseous (IO) UCB, D = IO UCB and IO MSC, F = IO UCB and IV MSC, and F = IV UCB and IO MSC.

Next, we correlated our flow cytometric analyses of hematopoietic recovery with direct cellularity assessment of the injected mouse bone marrow compartments (Figure 2). An analysis of the marrow cellularity by an un-biased hematopathologist revealed a myeloid-to-erythroid ratio of roughly 10:1 in all the mouse cohorts (Table 2). Interestingly, although statistically non-significant, the number of megakaryocyte per high-powered field was highest in the cohort with IV co-infused UCB and MSC. The overall bone marrow cellularity varied widely between cohort samples, and no significant differences were detected among cohorts (Table 2). These morphological data did not directly correlate with the numerical analysis of hematopoietic and immune reconstitution within the bone marrow samples by the more quantitative flow cytometry analysis.

## Discussion

In our current study, we demonstrated that IO co-transplantation of CD34-selected UCB and MSCs improved human hematopoietic cell engraftment over UCB controls administered IV. Interestingly, we also observed a predominance of early B-cell engraftment (CD19) within the bone marrow. Our observation is in agreement with previous published reports of human UCB engraftment in NOD/SCID mice [21,22]. It is not surprising that co-localizing MSCs and UCB within the bone improves engraftment, as MSCs support hematopoiesis through a number of mechanisms within the bone marrow niche. These mechanisms include cytokine support [16,17], especially within the perivascular space within the marrow where MSCs secrete CXCL12 and stem cell factor [18]. Furthermore, the injected MSC may have a role in replacing damaged or ineffective marrow resident MSCs. Our data demonstrating that IO co-administration of UCB and MSC resulted in a significant increase in both the CD19^+^ B-cell and CD3^+^ T-cell populations within the bone marrow suggest that MSCs, especially when directly co-infused into the bone marrow niche with UCBs, allow for a more robust immunologic re-constitution of the HSCs from UCB. Potential mechanisms by which MSC accomplish this is to act through both soluble factors, such as cytokines and growth factors, as well as via direct cell-cell contact with HSCs and cells of the bone marrow niche [23,24]. Direct contact of HSC by MSC in *ex vivo* expansion studies have demonstrated an increase in early lymphoid progenitors production [25,26]. Indeed, the importance of MSC support and interaction with the hematopoietic stem cell may be exemplified by the similar engraftment rate of the IO UCB cohort compared to the IV UCB cohort. The specific interaction between MSC and UCB in the bone marrow is not addressed in our study, and future investigations in this area may yield further scientific and mechanistic insights.

We did not observe a significant difference in CD45^+^, CD3^+^, CD13^+^, CD14^+^ or CD19^+^ cells in the peripheral blood among any of our cohorts at week 3 and week 6. However, human bone marrow engraftment in the absence of peripheral engraftment is considered an adequate surrogate marker in mice models [22].

Interestingly, a detailed histological analysis of the injected bone marrow did not demonstrate any differences in overall cellularity, megakaryocytic: erythrocytic (M:E) ratio, or the percentages of megakaryocytes among cohorts. This may be due to autologous recovery of murine bone marrow cells, which accompanied human UCB HSC engraftment. It is noteworthy that in the histologic evaluation of the IO MSC and IO UCB cohort, we observed an abundance of stromal tissue within the bone marrow microenvironment (Figure 2).

Overall, our results support and form the scientific basis for a new method of UCB transplantation that can be translated into clinical practice. UCB represents an important graft source for roughly one third of potential SCT patients who do not have a HLA matched related or unrelated donor [27,28]. However, UCB as a HSC source has its limitations, including lower cells dose resulting in slow or poor engraftment [1,29,30]. Various methods to optimize clinical UCB engraftment have been tried including *ex-vivo* expansion of UCB, intra-osseous transplantation of UCB, and intravenous co-transplantation of UCB and MSCs [12,20,31]. In addition, it has been demonstrated in patients that direct IO transplantation of a single UCB unit is safe, effective, and well tolerated following non-myeloablative conditioning preparatory regimen [31,32]. As a direct translation of our study presented here, we have now opened a clinical trial utilizing direct IO co-infusion of UCB and allogeneic MSC at the Seidman Cancer Center of the University Hospitals Case Medical Center (NCT02181478).

## Figures and Tables

**Table 1:** Summary of the percent CD45, CD3, CD13, CD14, CD19 in the right tibia bone marrow at 6 weeks post umbilical cord blood infusion.

	IV UCB	IO UCB	IV UCB + IO MSC	IV UCB + IV MSC	IO UCB + IV MSC	IO UCB + IO MSC
CD45	2.9%	2.5% (p > 0.05)	7% (p > 0.05)	22% (p < 0.01)	31% (p < 0.01)	34% (p < 0.01)
CD3	0.1%	0.2% (p > 0.05)	0.2% (p > 0.05)	0.5% (p > 0.05)	1.4% (p > 0.05)	3% (p < 0.05)
CD13	0.3%	0.7% (p > 0.05)	2.6% (p > 0.05)	0.3% (p > 0.05)	4.8% (p > 0.05)	4.7% (p > 0.05)
CD14	0.4%	0.2% (p > 0.05)	0.3% (p > 0.05)	0.5% (p > 0.05)	0.2% (p > 0.05)	0.9% (p > 0.05)
CD19	2.0%	0.9% (p > 0.05)	4% (p > 0.05)	21% (p < 0.01)	3% (p > 0.05)	28% (p < 0.01)

IV UCB = intravenous umbilical cord blood, IO UCB = intra-osseous umbilical cord blood, IV UCB + IV MSC = intravenous umbilical cord blood and mesenchymal stromal cells, IO UCB + IO MSC = intra-osseous umbilical cord blood and mesenchymal stromal cells, IV UCB + IO MSC = intravenous umbilical cord blood and intra-osseous mesenchymal stromal cells, IO UCB + IV MSC = intra-osseous umbilical cord blood and INTRAVENOUS mesenchymal stromal cells. p-values as compared to the intravenous umbilical cord blood group.

**Table 2:** Tabulation of histologic analysis from murine cohorts.

	IV UCB	IO UCB	IV UCB + IO MSC	IV UCB + IV MSC	IO UCB + IV MSC	IO UCB + IO MSC
**Megakaryocyte per high power field**	1.2	1.3	0.9	2.3	2.2	0.4
**Myeloid –to-erythroid Ratio**	10:1	10:1	10:1	10:1	10:1	10:1
**Cellularity**	62%	68%	82%	50%	46%	47%

IV UCB = intravenous umbilical cord blood, IO UCB = intra-osseous umbilical cord blood, IV UCB + IV MSC = intravenous umbilical cord blood and mesenchymal stromal cells, IO UCB + IO MSC = intra-osseous umbilical cord blood and mesenchymal stromal cells, IV UCB + IO MSC = intravenous umbilical cord blood and intra-osseous mesenchymal stromal cells, IO UCB + IV MSC = intra-osseous umbilical cord blood and intravenous mesenchymal stromal cells.

## Abbreviations and symbols

MSC: Human mesenchymal stromal cells
HSC: Hematopoietic stem cells
UCB: Umbilical cord blood
IO: Intra-osseous
IV: Intravenous
NSG: Non-obese diabetic severe combined immunodeficiency- IL2Rgammanull
SCT: Stem cell transplant
mAbs: Monoclonal antibodies
PBS: Phosphate Bufered Saline
HAS: Human Serum Albumin
IACUC: Institutional Animal Care and Use Committee
SEM: Standard error of the mean
